# Cell Responses to Calcium- and Protein-Conditioned Titanium: An In Vitro Study

**DOI:** 10.3390/jfb14050253

**Published:** 2023-05-01

**Authors:** Qiang Zhi, Yuehua Zhang, Jianxu Wei, Xiaolei Lv, Shichong Qiao, Hongchang Lai

**Affiliations:** 1 Department of Implant Dentistry, Shanghai Ninth People’s Hospital, College of Stomatology, Shanghai Jiao Tong University School of Medicine, Shanghai 200011, China; 2 National Clinical Research Center for Oral Diseases, Shanghai 200011, China; 3 Shanghai Key Laboratory of Stomatology, Shanghai Research Institute of Stomatology, Shanghai 200125, China; 4 Department of Orthodontics, Shanghai Ninth People’s Hospital, College of Stomatology, Shanghai Jiao Tong University School of Medicine, Shanghai 200011, China

**Keywords:** titanium, dental implant, bacterial infection, protein adsorption, peri-implantitis

## Abstract

Dental implants have become the leading choice for patients who lose teeth; however, dental implantation is challenged by peri-implant infections. Here, calcium-doped titanium was fabricated by the combinational use of thermal evaporation and electron beam evaporation in a vacuum; then, the material was immersed in a calcium-free phosphate-buffered saline solution containing human plasma fibrinogen and incubated at 37 °C for 1 h, creating calcium- and protein-conditioned titanium. The titanium contained 12.8 ± 1.8 at.% of calcium, which made the material more hydrophilic. Calcium release by the material during protein conditioning was able to change the conformation of the adsorbed fibrinogen, which acted against the colonization of peri-implantitis-associated pathogens (*Streptococcus mutans*, UA 159, and *Porphyromonas gingivalis*, ATCC 33277), while supporting the adhesion and growth of human gingival fibroblasts (*h*GFs). The present study confirms that the combination of calcium-doping and fibrinogen-conditioning is a promising pathway to meeting the clinical demand for suppressing peri-implantitis.

## 1. Introduction

Tooth loss has become an urgent problem for the aged population of China and the rest world [1], and dental implantation is now the leading choice for partial and full edentulism restoration [2]. However, the practice of dental implants is seriously affected by peri-implant infections, which are characterized by peri-implant mucositis and alveolar bone resorption, leading to implant loosening and shedding after osseointegration [2]. The prevalence of peri-implantitis for dental implants serving over 5 and 10 years is 9.6% and 26%, respectively [3]. In the Swedish population, the incidence of peri-implantitis can be even high up to 45% nine years after prosthodontics [4]. Bacterial colonization is a principal causal factor in the development of peri-implant infections, and the microbiota In peri-implantitis appears to be different from periodontal diseases. Higher counts of *Streptococcus mutans* and *Porphyromonas gingivalis* have been reported in peri-implantitis compared with periodontitis [5,6,7]. It is believed that the lack of a “biological seal” around the implants’ neck allows greater potential for bacterial colonization and peri-implant infections [8,9]. Therefore, improvement of the implant–fibroblast interactions in the transgingival portion of dental implants is also needed to provide effective soft tissue sealing against peri-implantitis.

Various disinfection strategies have been developed in recent decades to deal with peri-implantitis. These include near-infrared light triggered nitric oxide release by N,N′-Di-sec-butyl-N,N′-dinitroso-1,4-phenylene diamine (BNN6)-loaded molybdenum disulfide nanoflowers [10]; the combinational effect of cathodic proton consumption and anodic calcium production aroused by silver/calcium (Ag/Ca) galvanics on titanium surfaces [11]; the rapid release of silver nanoparticles promoted by the double Schiff base bonds in mussel-inspired hydrogels [12]; the galvanic effect of silver nanoparticles embedded in titanium on the proton motive force in bacterial membranes [13]; the prolonged antibacterial efficacy via slow hydrolysis of the hemiaminal ether linkage in polymers and sustained release of antibacterial agents [14]; the lysis action of the Schottky barriers between silver nanoparticles and a titanium oxide support mediated by storage of the bacteria-extruded electrons during bacterial adhesion in the dark [15]; and immune-instructive pathways to restore the host’s capability in bacterial clearance [16]. However, most of these designs are using toxic materials (e.g., DNA damage was found after exposure of human mesenchymal stem cells to 0.1 μg/mL of silver nanoparticles for 1, 3, and 24 h [17]; the molybdenum released by molybdenum disulfide can cause damage to the plasma membrane and inhibit efflux pump activities in mammalian cells [18]; and nitric oxide can react with DNA via multiple pathways and cause considerable damage to human cells and tissues [19]), targeting the pathogenic bacteria directly, which probably impact the implants’ tissue integration because it induced undesired host responses.

The placement of a dental implant initiates numerous biochemical interactions between the implant surface and the peri-implant fluid or tissues. The first process is the wetting of blood, which serves as a source of active ingredients, such as growth factors, cytokines, and chemokines that guarantee proper tissue integration of the implant [20,21]. Therefore, the wetting of blood or its components on implant surfaces and the subsequent biological effects have become a major concern in the field of surgical procedures and implantology [22,23]. Very recently, Cao et al. demonstrated that non-bactericidal calcium could unlock the antimicrobial sequence in human fibrinogen adsorbing to titanium, leading to high inhibition efficacy against *Pseudomonas aeruginosa* [24]. Previously, calcium-doping was found to be superior in promoting bone tissue integration [25] and gingival sealing around titanium implants [26], both of which are important to the long-term stability of dental implants. More importantly, fibrinogen adsorption is an important process which mediates the adhesion and growth of cells to implant materials and is the major focus of the biomaterial communities [27,28]. However, previous studies demonstrated that fibrinogen adsorption enhanced the adhesion of *Pseudomonas aeruginosa* and *Staphylococcus epidermidis* on various biomaterials, including polyurethane, polyvinylchloride, glass, and titanium [29,30], which likely undermines the prevention of peri-implant infections. Since calcium is an essential metal to humans [31,32], and protein adsorption is an immediate phase during medical device implantations, the use of calcium for endowing implantable devices of antibacterial activity is promising for clinical applications. Nonetheless, clinical translation of such a design needs further efforts that include testing its effect on bacterial strains on mammalian cells specific to an “*intended use*”.

Accordingly, the objective of present study is to test the effect of calcium doping and protein conditioning on the adhesion of pathogens and human gingival fibroblasts contributing to peri-implantitis and biological sealing, respectively. The results demonstrated that calcium- and fibrinogen-conditioned titanium has apparent activity against *Streptococcus mutans* (*Sm*, UA 159) and *Porphyromonas gingivalis* (*Pg*, ATCC 33277) colonization, but possess no side effect on human gingival fibroblasts growth.

## 2. Materials and Methods

### 2.1. Material Preparation

**Figure 1:**
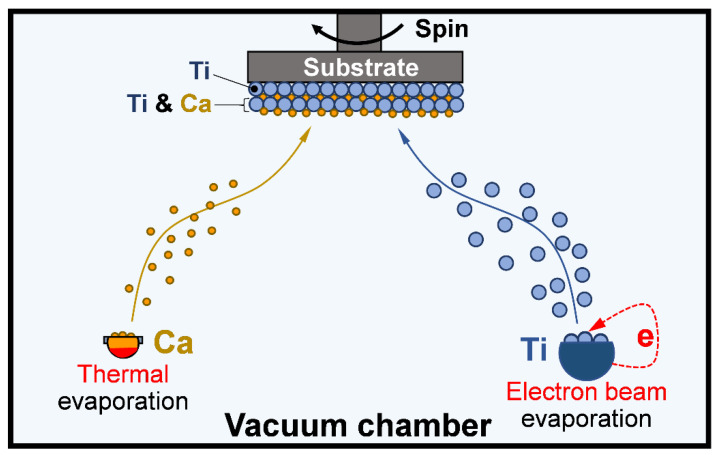
Illustrate the process for fabricating calcium-doped titanium by combining thermal evaporation and electron beam evaporation.

The pure titanium (Ti) and calcium-doped titanium (Ti-Ca) sample groups were fabricated by using a Leybold physical vapor deposition system (Univex 350, Köln, Germany), which is equipped with appendixes of thermal evaporation and an electron beam evaporation in a single vacuum chamber. As shown in Figure 1, the Ti samples were prepared by merely taking advantage of the electron beam evaporation appendix to source titanium (which has a purity of 99.99%, MaTeck Material Technologie and Kristalle GmbH, Germany), while the Ti-Ca samples were prepared by simultaneously using the thermal evaporation appendix to source calcium (which has a purity of 99.5%, MaTeck Material Technologie anda Kristalle GmbH, Jülich, Germany) and the electron beam evaporation system to deposit titanium on the substrates. The evaporation rate and the film thickness were kept at 0.3 nm/s and 200 nm, respectively. BOROFLOAT^®^ B33 glass discs (Jena 4 H Engineering GmbH, Jena, Germany) with a diameter of 15 mm were used as substrates to support the deposited materials, i.e., titanium and calcium-doped titanium (Figure 1).

### 2.2. Material Characterization

**Atomic Force Microscopy (AFM):** A Dimension 3100 AFM system (Digital Instruments, Santa Barbara, CA, USA) was employed to examine the surface morphology of the Ti and Ti-Ca samples. The AFM system is equipped with a standard Si_3_N_4_ tip on a cantilever beam.

**X-ray photoelectron spectroscopy (XPS)**: A Quantum 2000 XPS system (PHI Co., Chanhassen, MN, USA) was used to determine the chemical states of associated constituents in the material surface. The XPS system was excited with a monochromatic Al Kα source (1486.6 eV). The Multipak software supplied by the manufacturer was used for data analysis.

**Contact angle**: The Ti and Ti-Ca samples’ wettability was measured using an SL200B system (Solon, Shanghai, China). A suspended water droplet (1 μL) was advanced toward the sample surfaces by using a microliter syringe; then, the water-material contact images were recorded by a camera and analyzed with the manufacturer-supplied software. Every group was tested three times. Statistical analysis was conducted by using the one-way analysis of variance, and all the results were presented as the mean ± standard deviation.

**Inductively-coupled plasma optical emission spectrometry** (ICP-OES): The samples were immersed in 10 mL calcium-free phosphate-buffered saline solution (PBS) and incubated at 37 °C for 4 h, 1 day, 3 days, 7 days, 14 days, and 28 days. The PBS was refreshed at each time point and the obtained solutions were analyzed by inductively-coupled plasma optical emission spectrometry (ICP-OES) to determine the calcium released to the solutions by the samples (in mg/L/cm^2^). Every group was tested three times. Statistical analysis was conducted by using the one-way analysis of variance, and all the data were presented as the mean ± standard deviation.

**Fourier-transform infrared spectroscopy (FTIR)**: The protein-conditioned samples (Ti-F and Ti-Ca-F) were further examined by an ALPHA-P FTIR system (Bruker, Ettlingen, Germany). The FTIR spectra were recorded in a range of 4000–400 cm^−1^ with a resolution of 8 cm^−1^. The amide I and II regions of the spectra acquired from the Ti-Ca-F group were further analyzed by using the Fityk 1.3.1 software with a fixed half-maximum of 15.5 cm^−1^.

### 2.3. Protein Conditioning

The pure titanium and calcium-doped titanium were placed in 24-well plates and rinsed with calcium-free phosphate-buffered saline solution (PBS, preheated at 37 °C) twice; then, 2.5 mL of human plasma fibrinogen (HPF, Calbiochem, EMD Chemicals, Boston, MA, USA) contained phosphate-buffered saline solution (calcium-free, HPF concentration of 1 mg/L) was added to each well. The samples together with the HPF solution were incubated at 37 °C for 1 h. After that, the samples were rinsed with calcium-free PBS (preheated at 37 °C) once and pure water (preheated at 37 °C) twice, then dried at room temperature. As a result, fibrinogen-conditioned pure titanium (Ti-F) or calcium- and fibrinogen-conditioned titanium (Ti-Ca-F) were prepared for further studies.

### 2.4. Responses of Mammalian Cells

Human gingival fibroblasts (*h*GFs) were obtained from gingival tissue during the dental implantation from patients aged 18 to 40 years at Shanghai Ninth People’s Hospital, affiliated with Shanghai Jiaotong University School of Medicine, and all patients provided written informed consent (approved by the Medical Ethics Committee of the Ninth People’s Hospital, affiliated with Shanghai Jiao Tong University School of Medicine).

The dermal tissue without epithelial layer was cut into tiny pieces and stored in Dulbecco’s Modified Eagle’s Medium (Thermo Fisher Scientific, Waltham, MA, USA) with 20% fetal bovine serum (Hyclone, Logan, UT, USA) and 1% penicillin/streptomycin (Gibco, Waltham, MA, USA) with 5% CO_2_ and 95% air at 37 °C. Cells were passaged by trypsinization and *h*GFs from the fourth passage were used in this study.

The concentration of the suspension of the *h*GFs was adjusted to 1 × 10^4^ cells/mL and then l.0 mL cell suspension was seeded on the materials. After incubating at 37 °C for various durations (three samples per each group for every time point), i.e., 24 h, 72 h, and 168 h, the samples together with the adherent *h*GFs were rinsed with PBS three times and fixed with 4% paraformaldehyde for 10 min. Then the adherent cells on those samples were permeabilized with 0.5% Triton X-100 for 5 min. After that, the samples were stained with 200 μL (100 nM) phalloidin conjugated fluorescein isothiocyanate (FITC-Phalloidin, green color, Sigma, Livonia, MI, USA) for 1 h and 200 μL (100 nM) 4′,6-diamidino-2-phenylindole (DAPI, blue color, Sigma, Livonia, MI, USA) for 30 s in the dark. Then, the morphologies of the *h*GFs cells were examined under a confocal laser scanning microscope (Zeiss LSM 800, Jena, Germany) to evaluate their responses to the calcium-doped and fibrinogen-conditioned materials.

### 2.5. Responses of Bacterial Cells

To prepare proper bacterial suspensions, a colony of *Streptococcus mutans* (*Sm*, UA159) was cultivated at 37 °C in the brain–heart infusion broth medium with 5% CO_2_; while a colony of *Porphyromonas gingivalis* (*Pg*, ATCC 33277) was grown in the brain–heart infusion broth medium with hemin (0.5 mg/mL) and menadione (10 mg/mL) in an atmosphere of 10% CO_2_, 10% H_2_, and 80% N_2_.

Then, the responses of the bacterial cells to the materials were evaluated by seeding 10^7^ CFU/mL bacterial cells on the materials and incubating them at 37 °C for 5 h (three samples per each group). After that, the samples were rinsed three times using PBS and stained with 500 μL of the combination dye of SYTO 9 and propidium iodide (PI, LIVE/DEAD BacLight bacteria viability kits; Thermo Fisher Scientific, Waltham, MA, USA) in the dark for 30 min. All the sample groups were examined with a confocal laser scanning microscopy (Zeiss LSM 800, Jena, Germany).

## 3. Results

### 3.1. Features of the Materials

**Figure 2:**
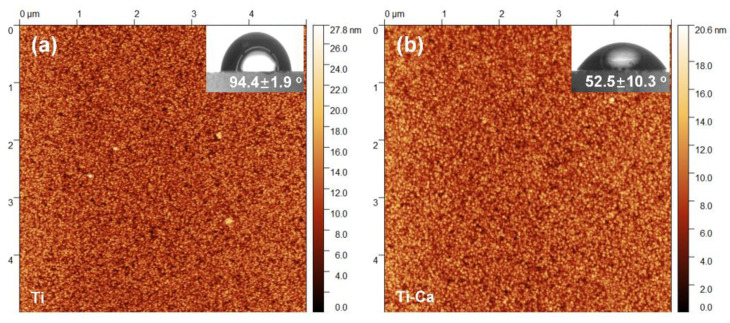
Atomic force microscopy (AFM) images of the pure titanium (**a**) and calcium-doped titanium (**b**) surfaces. The inserts are the corresponding water contact angle images.

The surface morphology of the materials was investigated by atomic force microscopy. As shown in Figure 2, both the pure titanium (Ti) and calcium-doped titanium (Ti-Ca) were composed of very small grains, whose size was in a range of 31–62 nm, forming a relatively smooth surface. The mean roughness (Sa) for the Ti and Ti-Ca groups was 2.1 ± 0.5 nm and 1.6 ± 0.1 nm, respectively. In addition, the maximum height (Sz) for the Ti and Ti-Ca groups was 22.8 ± 7.3 nm and 16.7 ± 3.6 nm, respectively. Although there is no significant difference in the surface roughness between the Ti and Ti-Ca groups, their wettability was significantly different. As shown by the inserts in Figure 2, the water contact angle for the Ti-Ca group was 52.5 ± 10.3 degrees, which was significantly lower than that of the Ti group (the record was 94.4 ± 1.9 degrees).

**Figure 3:**
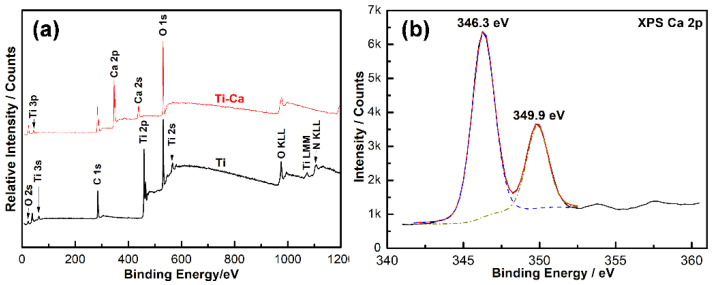
X-ray photoelectron spectroscopy (XPS) of the pure titanium and calcium-doped titanium surfaces: (**a**) XPS survey spectra and (**b**) XPS Ca 2p spectrum acquired from the calcium-doped titanium.

The surface chemistry of the materials was further checked by using X-ray photoelectron spectroscopy (XPS). As shown by the XPS survey spectra (Figure 3a), both the Ti and Ti-Ca groups were contaminated by carbon (C), oxygen (O), and nitrogen (N); while the calcium peaks were only sharply recorded in the Ti-Ca group. The atomic concentration of the doped calcium in the Ti-Ca group determined by XPS was 12.8 ± 1.8 at.%. A high-resolution Ca2p doublet at 349.9eV and 346.3 eV was identified as the Ti-Ca group (Figure 3b), which indicates the doped calcium was oxide [33]. The cumulative calcium concentration released by the Ti-Ca group was determined by inductively-coupled plasma optical emission spectrometry (ICP-OES). As shown in Table 1, the calcium-doped titanium was able to release 3.8 ± 0.1 mg/L/cm^2^ calcium, and this concentration increased to 9.7 ± 0.6 mg/L/cm^2^ as the incubation duration was prolonged to 28 days.

**Figure 4:**
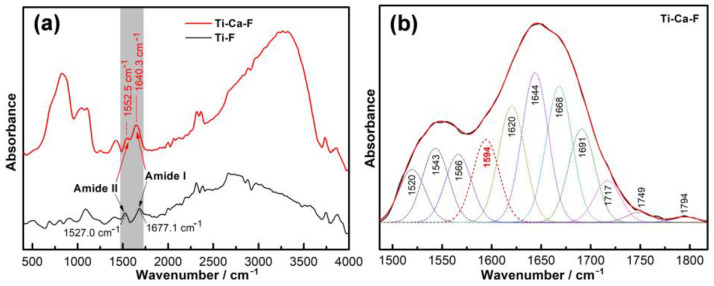
Fourier-transform infrared spectroscopy (FTIR) of the pure titanium and calcium-doped titanium surfaces pre-incubated in calcium-free PBS with 1 mg/L fibrinogen (**a**) and deconvolution of the amide band I and II regions of the FTIR spectrum acquired from Ti-Ca-F (**b**).

The materials fabricated by PVD were further conditioned by immersing in a calcium-free PBS containing 1mg/L of human plasma fibrinogen and incubated at 37 °C for 1 h. Then, both the protein-conditioned pure titanium (Ti-F) and calcium-doped titanium group (Ti-Ca-F) were examined by Fourier-transform infrared spectroscopy (FTIR). As shown by Figure 4a, the FTIR spectra of the fibrinogen’s amide I and II bands were sharply different. The absorbance peak of the amide I band for the Ti-F group was 1677.1 cm^−1^, which was over 36 wavenumbers higher than that of the Ti-Ca-F group (1640.3 cm^−1^). In addition, the absorbance peak of the amide II band for the Ti-F group was 1527.0 cm^−1^, which was over 25 wavenumbers lower than that of the Ti-Ca-F group (1652.5 cm^−1^). Moreover, the amide II and amide I bands (which correspond to the vibrations of in-plane N-H bending and C-N stretching [34]) for the Ti-Ca-F group overlaid each other, while those for the Ti-F group did not. In addition, a band at 1594 cm^−1^, arising from the stretching vibration of the -COO^−^ group [34], was identified in the deconvolution of the FTIR spectrum acquired from the Ti-Ca-F group (Figure 4b). These data indicated that the calcium released by the Ti-Ca tended to react with carboxyl groups and chelate with nitrogen in the adsorbed fibrinogen.

### 3.2. Bacterial Responses

**Figure 5:**
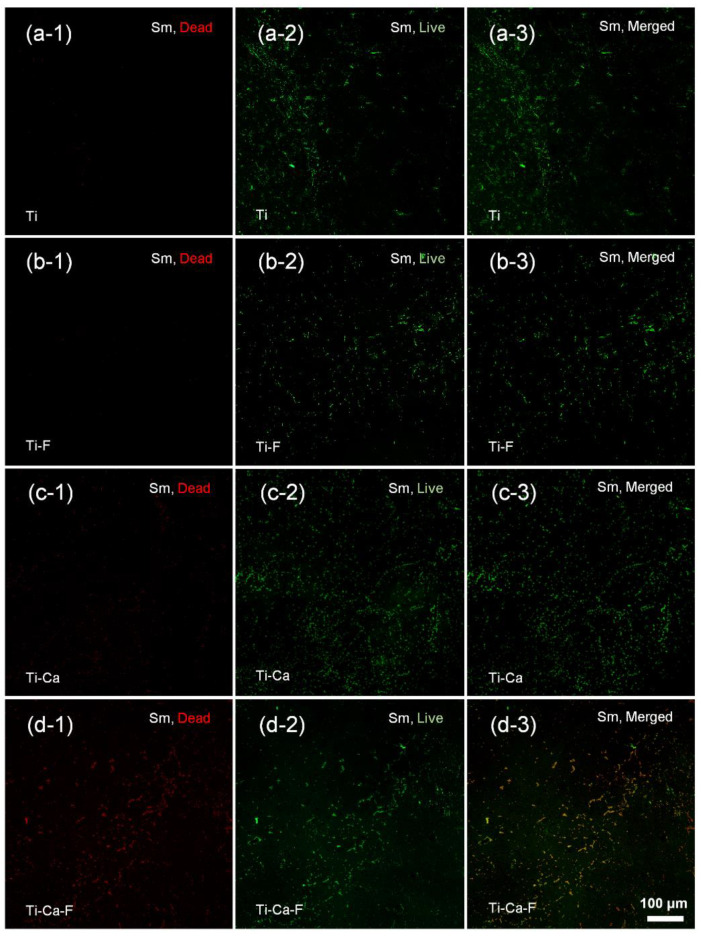
Fluorescent images of *Streptococcus mutans* (*Sm*, UA 159) cultured on pure titanium without (**a**-i) or with (**b**-i) protein conditioning; calcium-doped titanium without (**c**-i) or with (**d**-i) protein conditioning. (*i = 1, 2, 3 represent staining with dye of propidium iodide (PI, Red), SYTO 9 (Green), and the merged images*.)

**Figure 6:**
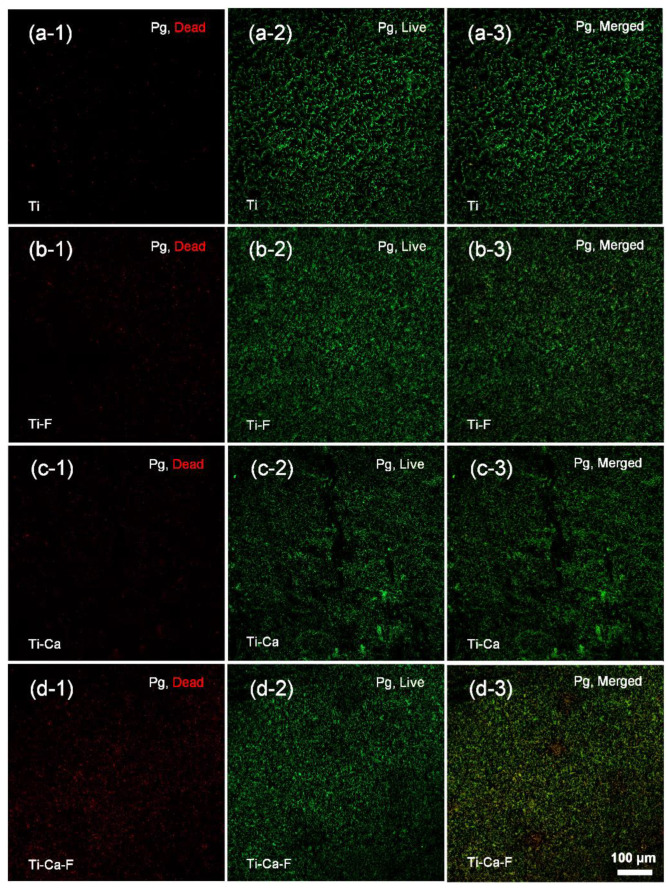
Fluorescent images of *Porphyromonas gingivalis* (*Pg*, ATCC 33277) cultured on pure titanium without (**a**-i) or with (**b**-i) protein conditioning; calcium-doped titanium without (**c**-i) or with (**d**-i) protein conditioning. (*i = 1, 2, 3 represent staining with dye of propidium iodide (PI, Red), SYTO 9 (Green), and the merged images*.)

The responses of pathogenic microbes associated with peri-implantitis were tested by seeding the *Streptococcus mutans* (UA 159) and *Porphyromonas gingivalis* (ATCC 33277) on the materials and incubated at 37 °C for 5 h. The bacterial colonization on the materials was evaluated by combinational staining with dye SYTO 9 (green, which indicates the total number of the adherent microbes) and propidium iodide (red, which indicates those bacteria of disintegrative membranes) and examined under a confocal laser scanning microscope. As to the *Streptococcus mutans* strain (Figure 5, fluorescent measurement was shown in Figure S1, Supplementary Materials), the intensity of green spots on Ti-Ca (Figure 5c-2) was stronger than that of the Ti (Figure 5a-2), Ti-F (Figure 5b-2), and Ti-Ca-F (Figure 5d-2) groups, which is consistent with previous reports that calcium doping itself facilitates bacterial colonization and growth [11,35]. Although the intensity of green spots on the Ti, Ti-F, and Ti-Ca-F groups was comparable, the intensity of the red spots on the Ti-Ca-F (Figure 5d-1) group is remarkably stronger than that of the Ti (Figure 5a-1), Ti-F (Figure 5b-1), and Ti-Ca (Figure 5c-1) groups, indicating that the Ti-Ca-F group has significant activity against the colonization of *Streptococcus mutans* strain. As to *Porphyromonas gingivalis* species (Figure 6, Fluorescent measurement was shown in Figure S2, Supplementary Materials), the intensity of green fluorescence in all the concerned groups is comparable (Figure 6a-2,b-2,c-2,d-2); whereas the intensity of red fluorescence on the Ti (Figure 6a-1), Ti-F (Figure 6b-1), and Ti-Ca (Figure 6c-1) groups was prominently weaker than that of the Ti-Ca-F (Figure 6d-1) group. These results demonstrate that the Ti-Ca-F group has stronger antibacterial activity compared with the pure titanium groups (both the Ti and Ti-F groups) and the calcium-doped titanium group without the condition of fibrinogen (Ti-Ca).

### 3.3. Response of the hGFs

**Figure 7:**
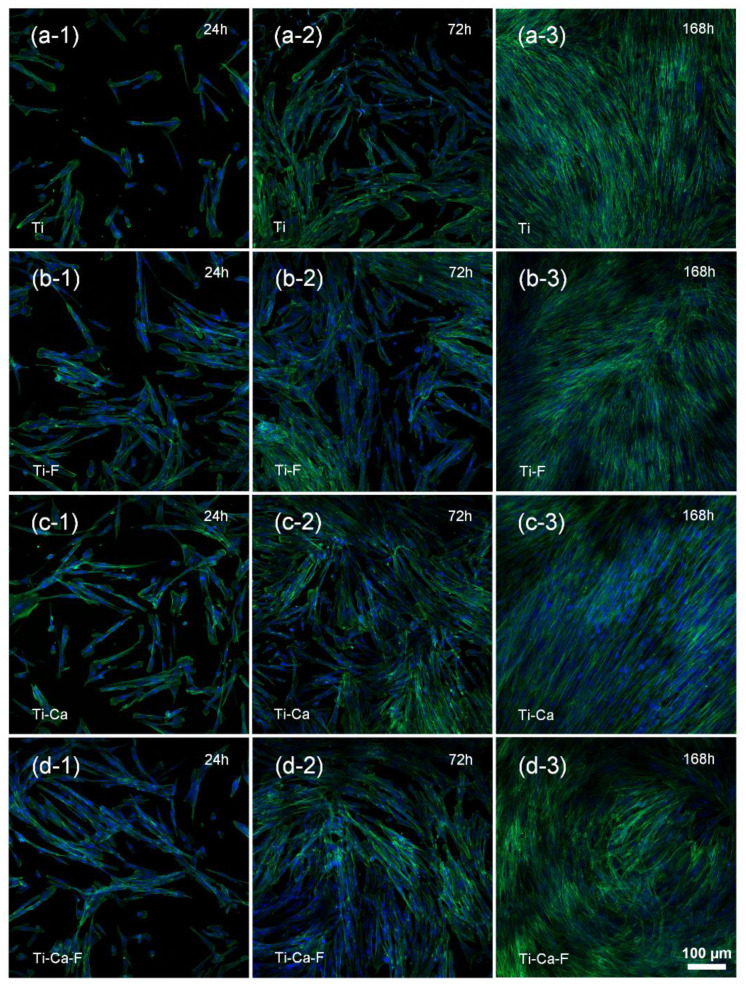
Fluorescent images (F-actin stained with FITC/green and nuclei stained with DAPI/blue) of human gingival fibroblasts cultured on pure titanium without (**a**-i) or with (**b**-i) protein conditioning; calcium-doped titanium without (**c**-i) or with(**d**-i) protein conditioning. (*i = 1, 2, 3 represent the culture duration of 24 h, 72 h, and 168 h, respectively*).

The responses of mammalian cells were tested by seeding human gingival fibroblasts on the materials and combinational staining with FITC (green, which shows the adhesion morphology of the cells) and DAPI (blue, indicates the corresponding cell nucleus); then, they were examined under a confocal laser scanning microscope after incubating at 37 °C for 24 h, 72 h, and 168 h. As shown in Figure 7 (cell number measurement was shown in Figure S3, Supplementary Materials), the fibroblasts on the Ti-F (Figure 7b-1), Ti-Ca (Figure 7c-1), and Ti-Ca-F (Figure 7d-1) groups were larger and spreading more rapidly than those on the Ti (Figure 7a-1) group after culturing the cells on the materials for 24 h. However, after culturing for 72 h, this difference in the growth of hGFs between Ti (Figure 7a-2), Ti-F (Figure 7b-2), Ti-Ca (Figure 7c-2), and Ti-Ca-F (Figure 7d-2) groups became weaker, all the sample groups demonstrated good support on the growth of hGFs (Figure 7a-3,b-3,c-3,d-3). These results demonstrated that the Ti-Ca-F group has good compatibility with the adhesion and growth of human gingival fibroblasts.

## 4. Discussion

Bacterial infection is a major complication associated with the practice of dental implants, and the development of safe and effective prevention strategies for peri-implantitis is a long-standing clinical demand in the community. Unlike most previous material designs targeting directly bacterial cells, calcium-doped titanium was very recently found capable of unlocking the activity of adsorbent fibrinogen against *Pseudomonas aeruginosa* colonization [24]. Since fibrinogen is a major player during the placement of implantable medical devices, this finding indicates a new paradigm for the development of implantable antibacterial surfaces. To verify the validity of this paradigm in dental implants, the present follow-up study tested the responses of peri-implantitis-associated pathogens, i.e., *Streptococcus mutans* (*Sm*, UA 159) and *Porphyromonas gingivalis* (*Pg*, ATCC 33277), and biological sealing-related human gingival fibroblasts to calcium- and fibrinogen-conditioned titanium.

The present study successfully doped 12.8 ± 1.8 at.% of calcium into titanium (Figure 3a) by combining thermal evaporation and the electron beam evaporation systems (Figure 1). The doped calcium was relatively easy to react with water, which makes the titanium surface more hydrophilic (the insert in Figure 2) and facilitates the release of calcium (Table 1). The released calcium tends to bind to fibrinogen, which was evidenced by a new FTIR band at 1594 cm^−1^ (Figure 4b). Moreover, the calcium in titanium was oxide because the metal is highly reactive and ready to react with the oxygen in the air (Figure 3b). This chemical feature of the doped calcium likely creates a basic local pH adjacent to the titanium surface and further facilitates the deprotonation of the amino acid in fibrinogen [36,37]. As a result, remarkable shifts of the amide II and amide I were detected by using FTIR (Figure 4a). These data demonstrated that the doped calcium changed the conformation of the adsorbed fibrinogen on titanium. This conformation change in fibrinogen endows the calcium- and protein-conditioned titanium (Ti-Ca-F) with activity against *Streptococcus mutans* and *Porphyromonas gingivalis* (Figure 5 and Figure 6), the pathogens associated with peri-implantitis, and good compatibility to the adhesion and growth of human gingival fibroblasts (Figure 7), which contribute to biological sealing.

The aforementioned antibacterial property and cytocompatibility of the calcium-doped and fibrinogen-conditioned titanium indicated a safe pathway toward solving the problem of peri-implant infections. A dental implant system has interfaces with both hard and soft tissue [38], which requires the dental implant surface to be compatible with or more soundly to promote the functions of both bone and gingival cells. Calcium has been proven effective in guaranteeing the integration of bone tissue and sealing of gum to titanium in vivo [25,26]; whereas previous studies found that calcium-doped titanium was insufficient to protect titanium from bacterial colonization in vitro [35,39], or that it even increased the adhesion of bacterial cells [40], which compromises the strategy of using calcium to build effective antibacterial titanium. The data in the present study also show that calcium doping itself has a minimal effect against the adhesion of both *Streptococcus mutans* and *Porphyromonas gingivalis* (Figure 5c-1 and Figure 6c-1), which is consistent with those reports. However, calcium doping in combination with fibrinogen adsorption was found to be effective against the colonization of the bacterial species highly associated with peri-implantitis (Figure 5d-1 and Figure 6d-1), which was likely because of the calcium-dependent conformation changes in the protein (Figure 4). The effect of calcium on the control of the structure of fibrinogen was well-known. It was demonstrated that the binding of calcium is usually mediated by the carboxyl residues in the protein [41]. The two αC regions interacted intramolecularly with each other and with the N-termini of Bβ chains of fibrinogen so that fibrinopeptide B could be removed upon switching these intramolecular interactions of αC regions to intermolecular manners [42]. Previous studies demonstrated that calcium at a higher concentration (about 0.05 M) could turn the carboxyl terminal regions of the Aα chains in fibrinogen from an inward position to a more solvent-exposed orientation, facilitating intermolecular interactions, and the calcium uptake by human fibrinogen was parallelly correlated with the release of the fibrinopeptide B [43,44]. In addition, the effect of calcium doping on changing the conformation of adsorbed fibrinogen was evidenced by the Fourier-transform infrared spectroscopy data (Figure 4) in our study. The removal of fibrinopeptide B likely unlocked the peptide Gly-His-Arg-Pro (Gly, glycine; His, histidine; Pro, proline; Arg, arginine) at the N-terminal end of the β chain, where the antibacterial peptide Bβ15-42 was located [45], further contributing to the prevention of the bacterial colonization as shown in Figure 5 and Figure 6.

Fibrinogen is a key player in reinforcing hemostasis and acts as the first line of host defense against bacterial infections. Virtually all tissue damages trigger local activation of the coagulation cascades, during which fibrinogen is transformed into fibrin, which accumulates outside the blood vessels within and around the impaired region of the tissue and physically prevents the invasion of pathogenic bacteria [46]. In addition, the efficacy of bacterial clearance within infected organs by the host innate immunity cells, i.e., neutrophils and macrophages, is also associated with fibrin depositions [47]. It was also well-established that fibrinogen adsorbs immediately to the surfaces of biomedical devices (or biomaterials) which come in contact with biological fluids during surgeries, and this determines the devices’ further interactions with immune effector cells and associated foreign body responses [27]. Unfortunately, uncontrolled adsorption of fibrinogen to biomaterials likely induces undesirable cell responses. Many previous studies have evidenced fibrinogen denaturation while adsorbing to titanium surfaces [48,49,50], and taking advantage of the semiconductive nature of previously non-stoichiometrical titanium oxide films was proposed to reduce such denaturation on titanium and subsequently improved compatibility [51,52]. In the present study, the activity against bacterial adhesion for the fibrinogen adsorbed on pure titanium (the Ti-F group, Figure 5b-1 and Figure 6b-1) was inferior to that adsorbed on calcium-doped titanium (the Ti-Ca-F group, Figure 5c-1 and Figure 6c-1). The inferiority of the Ti-F group against peri-implants-associated *Streptococcus mutans* and *Porphyromonas gingivalis* was probably the result of fibrinogen denaturation. As shown in Figure 4a, the conformation of the fibrinogen adsorbed on calcium-doped titanium (Ti-Ca-F) was different from that adsorbed on titanium.

Taken together, the data solidly confirm that the indirect antibacterial strategy originally proposed by Cao et al. [24], i.e., the combination of calcium-doping and fibrinogen-conditioning, is a promising strategy to prevent the colonization of pathogenic microbes and meet the clinical demand for suppressing peri-implantitis. The results obtained in our study demonstrated that antibacterial titanium implants do not need to target the bacterial cells directly, which may be a good pathway to designing and developing advanced surface modification processes that can properly balance the tissue integration and antimicrobial adhesion in implantable medical devices, especially dental implant systems. However, there are major limitations in the present study. The bacterial and mammalian cells’ adhesion to the materials is only considered in a short time. Since adsorbed fibrin(ogen) is likely degraded in normal healing processes, the effect of the adsorbed fibrinogen on cell adhesion should be checked for longer culture durations in future studies. Moreover, to promote the clinical translation of such a strategy, more efforts should be made. These include detailed examinations on the safety and efficacy of the disinfection of dental implants by calcium doping and protein conditioning *in vivo*.

## 5. Conclusions

In this study, calcium- and protein-conditioned titanium was fabricated first by a duplex deposition process composed of thermal evaporation deposition and electron beam evaporation deposition in a vacuum, and then by fibrinogen adsorption in a phosphate-buffered saline solution free of calcium. The responses of peri-implantitis and biological sealing associated cells were evaluated by seeding the cells on the materials and examining their morphology and growth behaviors. The results obtained in this study confirmed that calcium can regulate the conformation of the adsorbed fibrinogen, act against bacterial colonization (*Streptococcus mutans*, UA 159 and *Porphyromonas gingivalis*, ATCC 33277), and facilitate the adhesion and growth of human gingival fibroblasts, demonstrating that calcium- and protein-conditioning is a promising strategy for tackle peri-implant infections.

## Figures and Tables

**Table 1:** The cumulative release of calcium by the Ti-Ca group.

Time Point	Calcium Concentration (mg/L/cm^2^)
4 h	3.8 ± 0.1
1 day	4.2 ± 0.1
3 days	4.6 ± 0.1
7 days	5.6 ± 0.2
14 days	7.1 ± 0.5
28 days	9.7 ± 0.6

## Data Availability

The data presented in this study are available on request from the corresponding authors.

## Supplementary Materials

The following supporting information can be downloaded at: https://www.mdpi.com/article/10.3390/jfb14050253/s1, Figure S1: Fluorescent measurement (Integrated intensity) of the images was obtained by culturing Streptococcus mutans (UA 159) on the materials for 5 h (Figure 5). The RED BARS represent the results from images of the propidium iodide (PI, Red) channel, while the GREEN BARS represent the results from images of the SYTO 9 (Green) channel. The measurements were made by using ImageJ bundled with 64-bit Java 8. * *p* < 0.05; Figure S2. Fluorescent measurement (Integrated intensity) of the images obtained by culturing Porphyromonas gingivalis (Pg, ATCC 33277) on the materials for 5 h (Figure 6). The RED BARS represent the results from images of the propidium iodide (PI, Red) channel, while the GREEN BARS represent the results from images of the SYTO 9 (Green) channel. The measurements were made by using ImageJ bundled with 64-bit Java 8. * *p* < 0.05; Figure S3. The number of adherent fibroblasts on the materials (in an area of about 4.2 × 105 square microns). Cell counts were made according to fluorescent images (Figure 7), on which the cell nuclei were stained with DAPI/blue. * *p* < 0.05.
