# Supplementary material for: Cell Responses to Calcium- and Protein-Conditioned Titanium: An In Vitro Study
Source: J Funct Biomater. 2023 May 1;14(5):253. doi: 10.3390/jfb14050253 (PMC10218808; doi:10.3390/jfb14050253)
Supplement: Supplementary file 1 [file jfb-14-00253-s001.zip › jfb-2295232-supplementary.pdf]

Supplementary Materials

Cell responses to calcium- and protein-conditioned titanium: an in vitro study

Qiang Zhi[1] †, Yuehua Zhang[1] †, Jianxu Wei[1], Xiaolei Lv[1], Shichong Qiao[1,2] *, and Hongchang Lai[1,2] *

1 Shanghai Ninth People's Hospital, Shanghai Jiao Tong University School of Medicine; College of Stomatology, Shanghai Jiao Tong University; National Center for Stomatology; National Clinical Research Center for Oral Diseases; Shanghai Key Laboratory of Stomatology; Shanghai Research Institute of Stomatology, Shanghai 200125, China

2 Department of Implant Dentistry, Shanghai Ninth People's Hospital, Shanghai Jiao Tong University School of Medicine, Shanghai 200011, China

* Correspondence: shichong_qiao@139.com; lhc9@hotmail.com

† Qiang Zhi and Yuehua Zhang contributed equally to this manuscript.

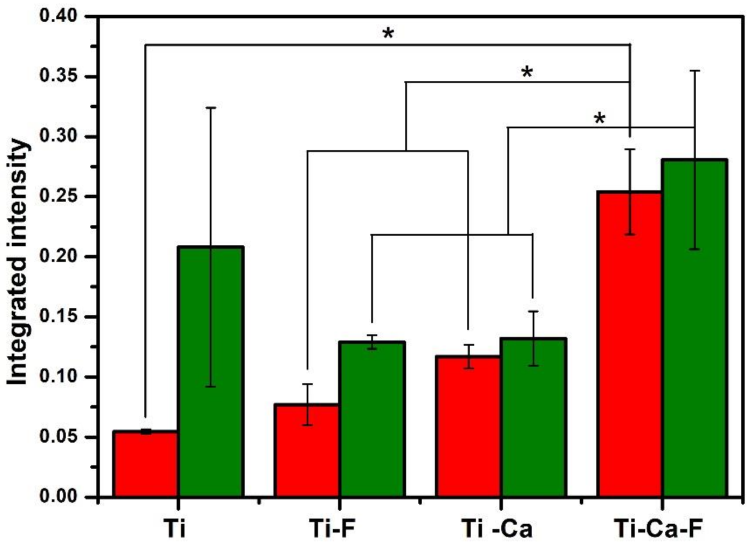

Figure S1. Fluorescent measurement (Integrated intensity) of the images was obtained by culturing *Streptococcus mutans* (UA 159) on the materials for 5 hours (Figure 5). The **RED BARS** represent the results from images of the propidium iodide (PI, Red) channel, while the **GREEN BARS** represent the results from images of the SYTO 9 (Green) channel. The measurements were made by using ImageJ bundled with 64-bit Java 8. * $p < 0.05$.

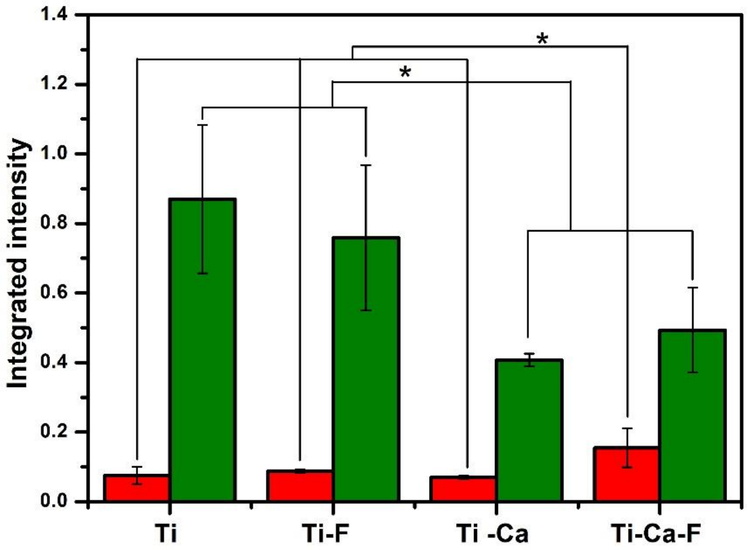

Figure S2. Fluorescent measurement (Integrated intensity) of the images obtained by culturing *Porphyromonas gingivalis (Pg,* ATCC 33277*)* on the materials for 5 hours (Figure 6). The **RED BARS** represent the results from images of the propidium iodide (PI, Red) channel, while the **GREEN BARS** represent the results from images of the SYTO 9 (Green) channel. The measurements were made by using ImageJ bundled with 64-bit Java 8. * $p < 0.05$.

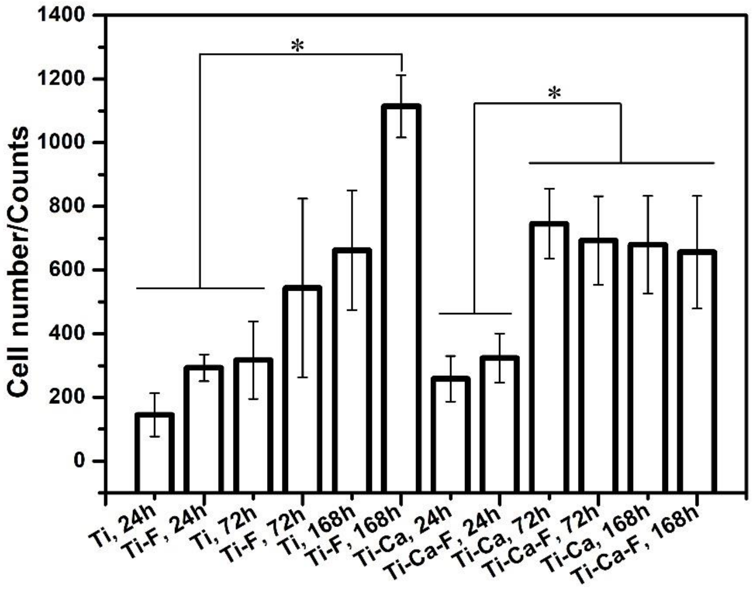

Figure S3. The number of adherent fibroblasts on the materials (in an area of about 4.2 x 10^5 square microns). Cell counts were made according to fluorescent images (Figure 7), on which the cell nuclei were stained with DAPI/**blue**. * $p < 0.05$.
